# Association of Body Roundness Index and A Body Shape Index with Obstructive Sleep Apnea: insights from NHANES 2015–2018 data

**DOI:** 10.3389/fnut.2024.1492673

**Published:** 2024-11-18

**Authors:** Xue Pan, Fang Liu, Jiayi Fan, Qihan Guo, Mengfei Guo, Yuxin Chen, Jingyao Sun, Xuezhao Cao

**Affiliations:** ^1^Department of Anesthesiology, The First Hospital of China Medical University, Shenyang, China; ^2^Department of Neurology, The First Hospital of China Medical University, Shenyang, China; ^3^Department of Clinical Medicine, China Medical University, Shenyang, China

**Keywords:** Body Roundness Index, A Body Shape Index, anthropometric indices, National Health and Nutrition Examination Survey, obesity, Obstructive Sleep Apnea

## Abstract

**Objective:**

This study examines the relationship between several anthropometric indices-Body Roundness Index (BRI), A Body Shape Index (ABSI), Waist-to-Weight Index (WWI), Waist Circumference (WC), and Body Mass Index (BMI)-and the prevalence of Obstructive Sleep Apnea (OSA) using data from the National Health and Nutrition Examination Survey (NHANES) spanning 2015 to 2018.

**Methods:**

A retrospective cross-sectional analysis of 7,004 adult participants was conducted using NHANES 2015–2018 data. Multivariable-adjusted logistic regression models were employed to assess the association between BRI, ABSI, and OSA. Non-linear relationships were explored via smooth curve fitting and threshold effect analysis using a two-part linear regression model. Subgroup analyses identified sensitive populations, and the discriminatory power of the indices in screening OSA was assessed using Receiver Operating Characteristic (ROC) curves.

**Results:**

The analysis revealed a significant positive association between BRI and OSA, with a threshold effect observed at a BRI of 4.3. Below this threshold, OSA risk increased with higher BRI; however, no significant association was found above this threshold. Similarly, ABSI demonstrated a threshold effect at 8.2, with OSA risk positively associated to the left and negatively associated to the right. Subgroup analyses indicated stronger associations in younger and non-diabetic populations. ROC analysis identified BRI as a promising predictive tool for OSA, with an AUC of 0.64 (95% CI: 0.62–0.65).

**Conclusion:**

BRI demonstrates significant potential as a predictive index for OSA incidence, warranting further large-scale prospective studies to validate these findings.

## 1 Introduction

Obstructive Sleep Apnea (OSA) is a common disorder characterized by repetitive episodes of upper airway obstruction during sleep, leading to intermittent hypoxemia and frequent arousals. A study published in *The Lancet* estimates that approximately 936 million adults worldwide, aged 30–69, suffer from mild to severe OSA, with 425 million experiencing moderate to severe forms of the condition (1). OSA is closely associated with several serious health complications, including hypertension (2), diabetes (3), stroke (4), and cognitive decline (5). The morbidity and mortality associated with OSA are largely attributable to the increased burden of cardiovascular disease, including arrhythmias (6), underscoring the importance of identifying new and more precise biomarkers for early OSA diagnosis.

Obesity is one of the strongest predictors of OSA, with weight loss often recommended as an initial treatment for patients with mild OSA (7, 8). Excessive fat deposition in the pharyngeal airway and central fat accumulation can contribute to airway narrowing, thereby increasing the likelihood of OSA (9–11). The relationship between obesity and OSA is further complicated by obesity-related hormones and cytokines, such as leptin, which may exacerbate respiratory instability during sleep (12). Notably, visceral fat, rather than total body fat, plays a pivotal role in the development of OSA, emphasizing the need for accurate assessment of fat distribution in evaluating OSA risk (13, 14).

Traditional obesity indices, such as Waist Circumference (WC) and Body Mass Index (BMI), have limitations in accurately predicting fat distribution, particularly visceral fat. To address these gaps, new indices such as the Body Roundness Index (BRI) and A Body Shape Index (ABSI) have been developed. The BRI, developed by Thomas et al. in 2013, integrates height and waist circumference to provide a more nuanced assessment of body fat distribution. Research has demonstrated that BRI more accurately reflects visceral fat and overall body fat percentage compared to conventional measurements like WC and BMI. Furthermore, recent studies have identified BRI as a predictor of various health conditions, including cardiovascular diseases, gallstones (15), hypertension (16), bone mineral density (17), and all-cause mortality (18). Conversely, the ABSI, proposed by Krakauer et al., has proven effective in predicting mortality independently of BMI in the U.S. population (19). It has also been significantly associated with various metabolic syndromes across multiple studies (20, 21). While both indices incorporate elements of WC and height, their applications differ. The BRI is primarily used to evaluate an individual’s overall physical fitness, whereas ABSI is more focused on identifying health risks associated with abdominal obesity. Despite their unique roles in assessing body composition, the relationship between BRI and ABSI and the prevalence of OSA remains largely unexplored.

This study aims to address this gap by investigating the association between BRI, along with other novel Anthropometric Indices (AHIs) like A Body Shape Index (ABSI), and the prevalence of OSA using data from the 2015–2018 National Health and Nutrition Examination Survey (NHANES). The hypothesis is that BRI is a strong predictor of OSA, and that managing AHIs could play a critical role in preventing and treating OSA. By leveraging the extensive and representative data from NHANES, this study seeks to provide valuable insights into the potential clinical utility of AHIs in the context of OSA.

## 2 Methods

### 2.1 Study design and population

**Figure 1:**
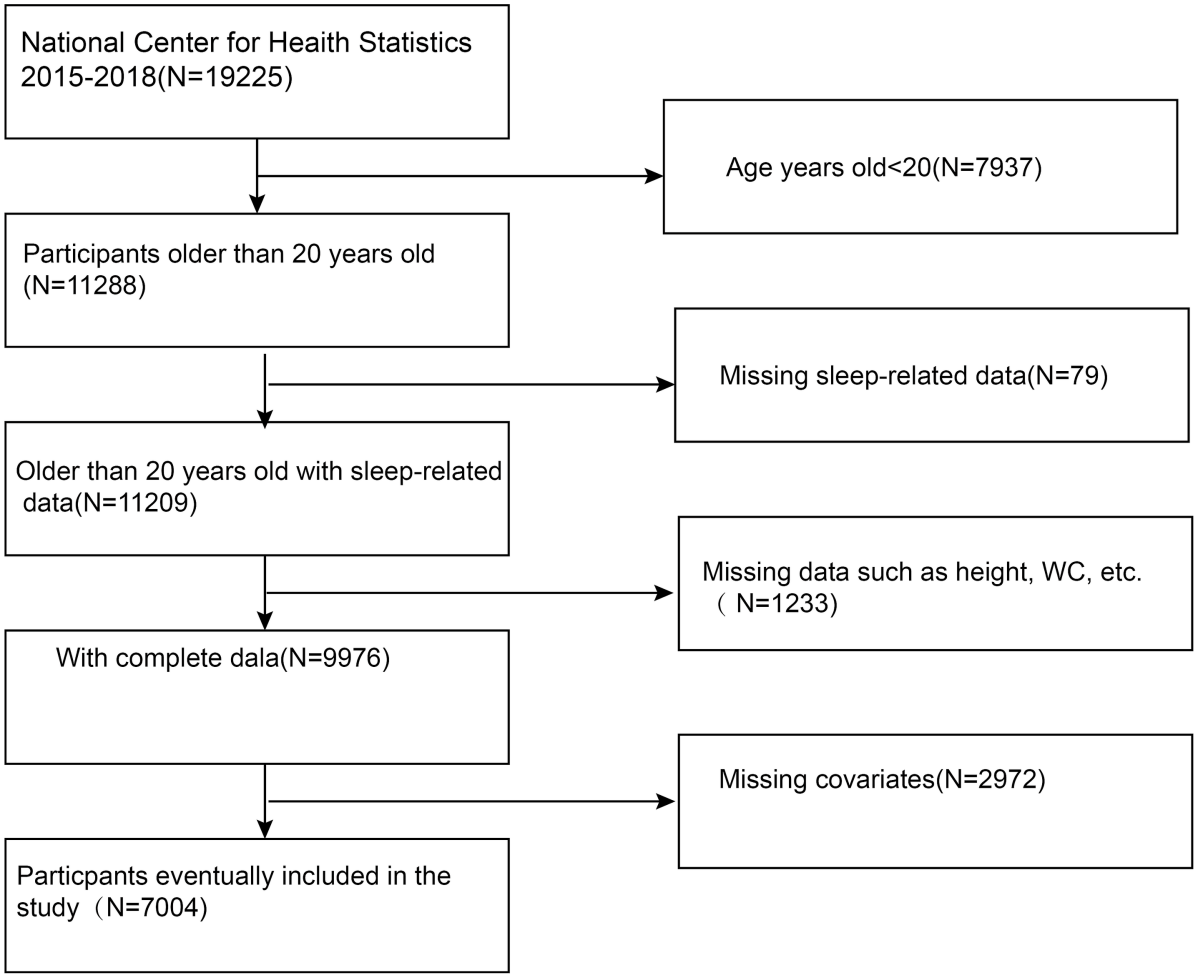
Flowchart of participant selection. WC, Waist Circumference.

This cross-sectional study sought to examine the relationship between the BRI and other novel anthropometric indices with OSA using data from the NHANES from 2015 to 2018. NHANES, managed by the National Center for Health Statistics (NCHS), employs a complex, stratified, multistage probability sampling design to generate a nationally representative sample of the U.S. population. Each survey cycle includes demographic data, physical measurements, laboratory tests, and dietary information. Detailed methodologies and documentation can be accessed at NHANES website. This study focused on participants aged 20 and older, excluding those with incomplete data on OSA, height, waist circumference, or other key variables. This study ultimately included 7,004 participants. The specific inclusion and exclusion criteria are detailed in Figure 1.

### 2.2 Calculation of anthropometric indices

The Body Roundness Index (BRI) was calculated using the following formula:


$$BRI=364.2-365.5\times {\left\{1-{\left(WC\left(\mathrm{cm}\right)/\left(2\pi \right)/\left(0.5\times \mathrm{height}\left(\mathrm{cm}\right)\right)\right)}^{2}\right\}}^{0.5}$$

A Body Shape Index (ABSI) was calculated based on WC, height, and weight using the formula:


$$\mathrm{ABSI}=WC\left(m\right)÷\left(BMI{\left(\mathrm{kg}/m^{2}\right)}^{\frac{2}{3}}\times \mathrm{Height}{\left(m\right)}^{\frac{1}{2}}\right)$$

The Waist-to-Weight Index (WWI) was calculated by dividing WC (in centimeters) by the square root of body weight (in kilograms).


$$WWI=WC\left(\mathrm{cm}\right)÷\surd \left(\mathrm{Weight}\left(\mathrm{kg}\right)\right)$$

### 2.3 Diagnosis of probable OSA

Probable OSA (pOSA) was identified based on participants’ responses to three dichotomous questions in the NHANES survey. Participants were classified as having pOSA if they answered “Yes” to at least one of the following questions (22, 23):

1. “Despite sleeping approximately 7 h or more per night, do you feel excessively sleepy during the day 16–30 times a month on weekdays or workdays?”
2. “Do you experience gasping for breath, snoring, or stopping breathing 3 or more nights per week?”
3. “Do you snore 3 or more nights per week?”

### 2.4 Covariates

To adjust for potential confounding factors, several covariates were included in the multivariable-adjusted models. These covariates included demographic variables (age, gender, race/ethnicity, marital status, education level, and poverty income ratio), lifestyle factors (smoking status and alcohol consumption), and health conditions (hypertension, diabetes, and history of cardiovascular disease) (Supplementary Table 1). Detailed measurement methods for these variables are available on the CDC’s NHANES website: www.cdc.gov/nchs/nhanes/.

### 2.5 Statistical analysis

Weighted statistical analyses were performed for each participant according to the sample weighting associated with NHANES’s intricate multistage cluster sampling design. Group differences were assessed using chi-square test for categorical variables and analysis of variance (ANOVA) for continuous variables. To assess the relationship between BRI and pOSA, multivariable logistic regression models were utilized. Three models were applied: Model I with no covariates adjusted, Model II adjusted for age, gender, and race/ethnicity, and Model III further adjusted for marital status, education level, Poverty Income Ratio (PIR), smoking status, alcohol use, hypertension, cardiovascular disease, and history of diabetes. Subgroup analyses were conducted by stratifying the population based on gender, age, and other key variables.

Smoothing curve fitting was employed to detect nonlinear relationships between pOSA and the indices. A two-segment linear regression model was then employed to investigate threshold effects. The predictive power of BRI, ABSI, and other indices for pOSA was assessed using the corresponding Area Under Curve (AUC) and Receiver Operating Characteristic (ROC) curves. Statistical analyses were performed using R software (version 4.0.5) and EmpowerStats (version 2.0). Sample interview weights were applied to adjust for the survey design, and a *p*-value of <0.05 was considered statistically significant.

## 3 Results

### 3.1 Baseline characteristics of participants

The study included 7,004 participants with a mean age of 48.27 ± 17.30 years (Figure 1). Of these participants, 49.30% were male and 50.70% were female. The mean value of BRI index was 2.16 ± 1.20. The overall prevalence of pOSA in the cohort was 49.39%. BRI quartiles were categorized as follows: ≤1.32 (Quartile 1), 1.33–1.98 (Quartile 2), 1.99–2.80 (Quartile 3), and > 2.80 (Quartile 4). Participants in Quartile 4 of the BRI tended to be older, had a higher proportion of females, lower PIR, lower educational attainment, and exhibited higher rates of smoking, alcohol consumption, hypertension, diabetes, cardiovascular disease, and pOSA compared to those in Quartile 1. Table 1 provides a detailed overview of the weighted demographic baseline characteristics of the study participants.

**Table 1:** Baseline characteristics of participants in the NHANES 2015–2018.

Variables	Quartiles of BRI				*p*-Value
	Q1	Q2	Q3	Q4	
Age (year)	39.06 ± 0.67	48.22 ± 0.62	50.62 ± 0.59	50.06 ± 0.74	<0.0001
BMI (kg/m^2^)	22.59 ± 0.11	27.04 ± 0.12	30.99 ± 0.12	38.54 ± 0.22	<0.0001
PIR	3.18 ± 0.08	3.29 ± 0.07	3.12 ± 0.09	2.93 ± 0.09	0.0088
Gender (%)					<0.0001
Male	49.22	55.73	54.08	36.15	
Female	50.78	44.27	45.92	63.85	
Race (%)					<0.0001
Mexican American	5.56	8.25	11.84	11.12	
Other Hispanic	4.82	7.17	7.34	5.83	
Non-Hispanic White	66.11	65.86	63.53	65.58	
Non-Hispanic Black	12.45	8.07	9.27	12.19	
Other races	11.06	10.66	8.01	5.28	
Educational level (%)					<0.0001
Under high school	7.68	12.00	12.15	12.09	
High school or equivalent	19.01	21.14	26.41	25.97	
Above high school	73.31	66.86	61.43	61.94	
Marital status (%)					<0.0001
Married	59.17	68.04	67.17	61.26	
Single	10.76	17.90	18.64	21.79	
With partner	30.07	14.06	14.19	16.95	
Smoking status(%)					<0.0001
Never smoker	61.71	57.62	53.90	54.89	
Former smoker	18.44	22.65	28.95	29.27	
Current smoker	19.85	19.72	17.15	15.85	
Alcohol (%)					0.0105
Never	71.56	71.55	70.26	66.22	
Drinks	28.44	28.45	29.74	33.78	
Diabetes (%)					<0.0001
No	97.11	91.77	82.88	73.59	
Yes	2.89	8.23	17.12	26.41	
Hypertension (%)					<0.0001
No	79.07	66.22	63.06	54.15	
Yes	20.93	33.78	36.94	45.85	
CVD (%)					<0.0001
No	97.97	94.93	93.16	89.98	
Yes	2.03	5.07	6.84	10.02	
pOSA (%)					<0.0001
No	71.20	52.01	46.51	38.89	
Yes	28.80	47.99	53.49	61.11	

NHANES, National Health and Nutrition Examination Survey; BMI, Body Mass Index; PIR, Poverty Income Ratio; CVD, Cardiovascular Disease; pOSA, Probable Obstructive Sleep Apnea.

### 3.2 Association between AHIs and pOSA prevalence

BRI and WWI exhibited a more robust association with pOSA than other anthropometric measures, including ABSI, WC, and BMI. In the fully adjusted model, a positive association was observed between BRI and pOSA (OR 1.50; 95% CI: 1.42, 1.58; *p* < 0.0001), indicating that each unit increase in BRI corresponded to a 50% higher prevalence of pOSA in the subjects. Similarly, WWI was also positively correlated with pOSA (OR 1.51; 95% CI: 1.40, 1.63; *p* < 0.0001). The correlation between BRI and pOSA remained robust even when BRI was categorized into quartiles. Participants in Quartile 4 were 267% more likely to develop pOSA compared to those in Quartile 1. Notably, ABSI did not demonstrate a significant association with pOSA in the fully adjusted model (Table 2).

**Table 2:** Multivariable logistic regression models for the association between anthropometric indices and pOSA.

	OR (95% CI), *p*-value		
	Model I	Model II	Model III
pOSA			
BRI	1.49 (1.43, 1.56) <0.0001	1.51 (1.44, 1.58) <0.0001	1.50 (1.42, 1.58) <0.0001
ABSI	1.38 (1.25, 1.52) <0.0001	1.12 (1.00, 1.26) 0.0523	1.07 (0.94, 1.21) 0.3184
WC	1.07 (1.06, 1.08) <0.0001	1.08 (1.07, 1.08) <0.0001	1.07 (1.06, 1.08) <0.0001
BMI	1.03 (1.03, 1.04) <0.0001	1.03 (1.03, 1.03) <0.0001	1.03 (1.03, 1.03) <0.0001
WWI	1.46 (1.38, 1.54) <0.0001	1.53 (1.43, 1.64) <0.0001	1.51 (1.40, 1.63) <0.0001
Categories			
Quartile1	Ref	Ref	Ref
Quartile2	2.07 (1.81, 2.38) <0.0001	1.93 (1.67, 2.22) <0.0001	1.82 (1.57, 2.12) <0.0001
Quartile3	2.58 (2.25, 2.96) <0.0001	2.40 (2.07, 2.78) <0.0001	2.32 (1.98, 2.71) <0.0001
Quartile4	3.72 (3.23, 4.27) <0.0001	3.85 (3.31, 4.47) <0.0001	3.67 (3.12, 4.32) <0.0001

OR, Odds Ratio; CI, Confidence Interval; pOSA, Probable Obstructive Sleep Apnea; BRI, Body Roundness Index; ABSI, A Body Shape Index; WC, Waist Circumference; BMI, Body Mass Index; WWI, Waist-to-Weight index.

### 3.3 Curve fitting and threshold effect analysis

**Figure 2:**
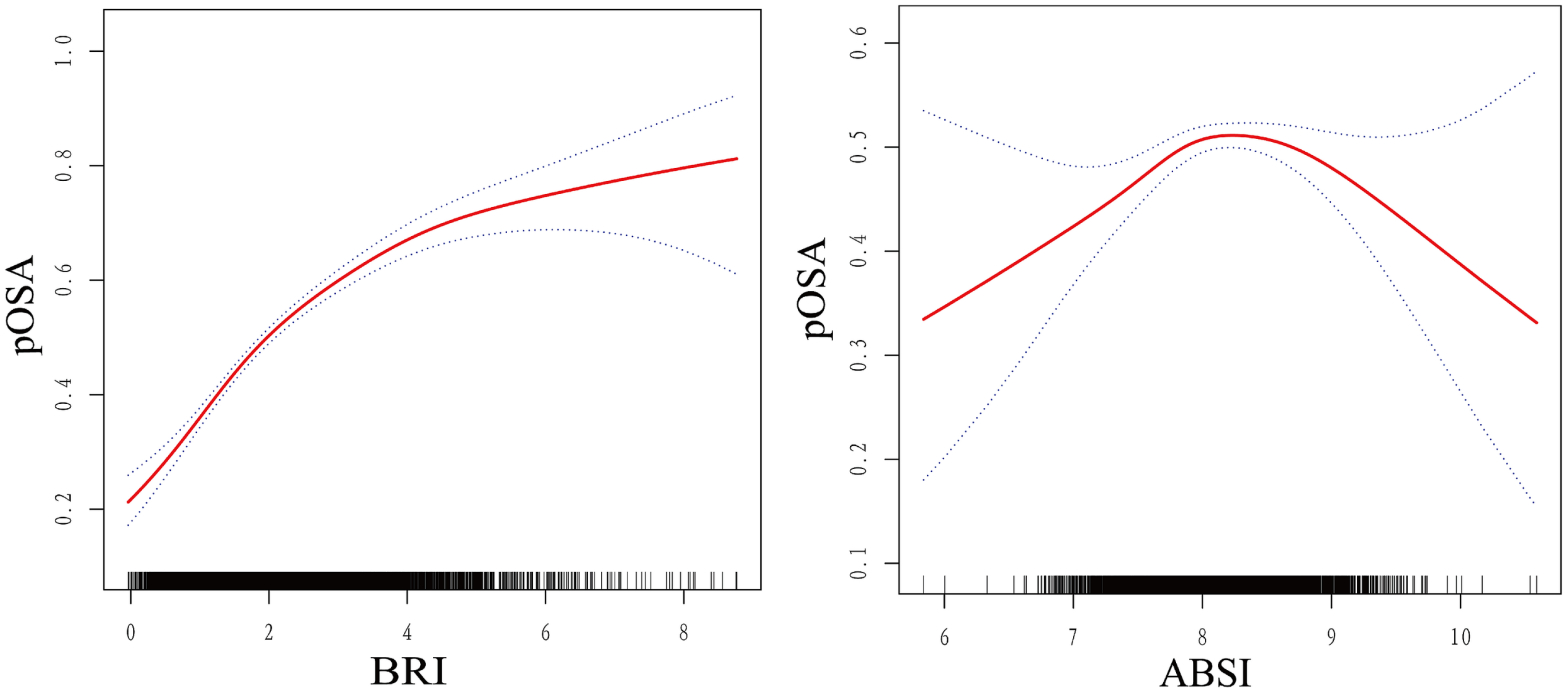
Smoothed curve fit between novel anthropometric indices and OSA. The blue bars show the fitted 95% confidence intervals (95% CIs) and the fitted smoothed curves are shown in red. pOSA, Probable Obstructive Sleep Apnea; BRI, Body Roundness Index; ABSI, A Body Shape Index; WC, Waist Circumference; BMI, Body Mass Index; WWI, Waist-to-Weight index.

The smoothed curve fitting results from Model 3 revealed a saturation effect in the relationship between BRI and pOSA prevalence. Specifically, the analysis indicated an inverted U-shaped curve between ABSI and pOSA, with a distinct inflection point (Figure 2). The smoothed curve fitting for the remaining indicators was similar to that of BRI index with pOSA (Supplementary Figures 1–3).

Threshold effect analysis identified an inflection point of 4.3 for BRI. Below this threshold, a significant association with pOSA prevalence was observed (OR 1.59; 95% CI: 1.50, 1.69; *p* < 0.0001). However, beyond this point, the association was not statistically significant (OR 1.02; 95% CI: 0.85, 1.24; *p* = 0.8076), with a log-likelihood ratio test yielding a *p*-value of <0.001 (Table 3). For ABSI, an inflection point of 8.2 was identified. The association with pOSA prevalence was positive below this inflection point (OR 1.40; 95% CI: 1.14, 1.73; *p* = 0.0013) and negative above it (OR 0.78; 95% CI: 0.62, 0.97; *p* = 0.0279), with a log-likelihood ratio test *p*-value of <0.001 (Table 4).

**Table 3:** The threshold effect analysis of the BRI on pOSA risk.

	OR (95% CI), *p*-value
Fitting by the standard linear model	1.50 (1.42, 1.58) <0.0001
Fitting by the two-piecewise linear model	
Inflection point	4.3
BRI < 4.3	1.59 (1.50, 1.69) <0.0001
BRI > 4.3	1.02 (0.85, 1.24) 0.8076
*p* for log-likelihood ratio	<0.001

OR, Odds Ratio; CI, Confidence Interval; pOSA, Probable Obstructive Sleep Apnea; BRI, Body Roundness Index.

**Table 4:** The threshold effect analysis of the ABSI on pOSA risk.

	OR (95% CI), *p*-value
Fitting by the standard linear model	1.07 (0.94, 1.21) 0.3184
Fitting by the two-piecewise linear model	
Inflection point	8.2
ABSI<8.2	1.40 (1.14, 1.73) 0.0013
ABSI>8.2	0.78 (0.62, 0.97) 0.0279
*p* for log-likelihood ratio	<0.001

OR, Odds Ratio; CI, Confidence Interval; pOSA, Probable Obstructive Sleep Apnea; ABSI, A Body Shape Index.

### 3.4 Subgroup analysis

Multiple subgroup analyses and interaction tests were performed to evaluate the stability of the BRI-pOSA relationship and to detect any subgroup variations (Table 5). The findings from these analyses revealed a consistent relationship between BRI and pOSA across most subgroups. Notably, age modified the relationship between BRI and pOSA prevalence (*p* for interaction <0.0001), highlighting the importance of monitoring BRI even in individuals under 60 years of age. Additionally, the relationship between BRI and pOSA varied within the diabetic population (*p* for interaction = 0.0057). While a significant correlation was observed in the diabetic group (OR 1.28; 95% CI: 1.14, 1.42; *p* < 0.0001), the relationship was even stronger in those without diabetes (OR 1.55; 95% CI: 1.47, 1.65; *p* < 0.0001).

**Table 5:** Stratified analysis of the correlation between BRI and pOSA.

Subgroup analysis	Foerst plot	pOSA, OR (95%CI), *p*-value	*p* for interaction
Age (year)	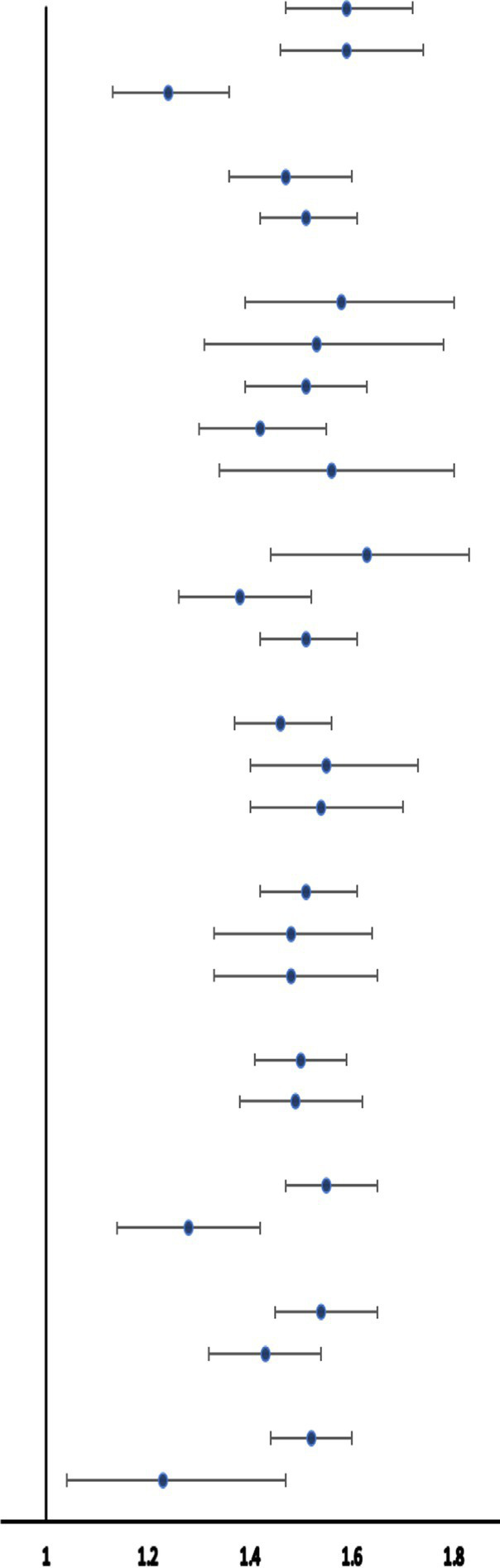		<0.0001
<=40		1.59 (1.47, 1.72) <0.0001	
>40, <=60		1.59 (1.46, 1.74) <0.0001	
>60		1.24 (1.13, 1.36) <0.0001	
Gender (%)			0.5998
Male		1.47 (1.36, 1.60) <0.0001	
Female		1.51 (1.42, 1.61) <0.0001	
Race (%)			0.6252
Mexican American		1.58 (1.39, 1.80) <0.0001	
Other Hispanic		1.53 (1.31, 1.78) <0.0001	
Non-Hispanic White		1.51 (1.39, 1.63) <0.0001	
Non-Hispanic Black		1.42 (1.30, 1.55) <0.0001	
Other races		1.56 (1.34, 1.80) <0.0001	
Educational level (%)			0.0883
Under high school		1.63 (1.44, 1.83) <0.0001	
High school or equivalent		1.38 (1.26, 1.52) <0.0001	
Above high school		1.51 (1.42, 1.61) <0.0001	
Marital status (%)			0.4957
Married		1.46 (1.37, 1.56) <0.0001	
Single		1.55 (1.40, 1.73) <0.0001	
With partner		1.54 (1.40, 1.70) <0.0001	
Smoking status (%)			0.9048
Never smoker		1.51 (1.42, 1.61) <0.0001	
Former smoker		1.48 (1.33, 1.64) <0.0001	
Current smoker		1.48 (1.33, 1.65) <0.0001	
Alcohol (%)			0.9258
Drinks		1.50 (1.41, 1.59) <0.0001	
Never		1.49 (1.38, 1.62) <0.0001	
Diabetes (%)			0.0057
No		1.55 (1.47, 1.65) <0.0001	
Yes		1.28 (1.14, 1.42) <0.0001	
Hypertension (%)			0.1025
No		1.54 (1.45, 1.65) <0.0001	
Yes		1.43 (1.32, 1.54) <0.0001	
CVD (%)			0.0017
No		1.52 (1.44, 1.60) <0.0001	
Yes		1.23 (1.04, 1.47) 0.0174	

OR, Odds Ratio; CI, Confidence Interval; pOSA, Probable Obstructive Sleep Apnea; BRI, Body Roundness Index; CVD, Cardiovascular Disease.

### 3.5 ROC curve analysis

**Figure 3:**
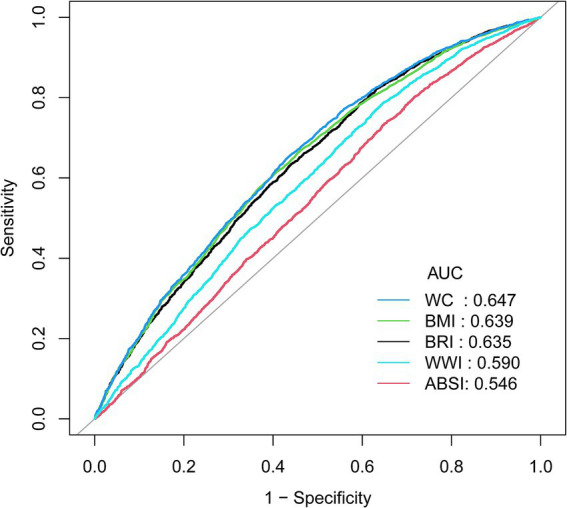
ROC curve between the AHIs with OSA. ROC, Receiver Operating Characteristic; AHIs, Anthropometric Indices; pOSA, Probable Obstructive Sleep Apnea; BRI, Body Roundness Index; ABSI, A Body Shape Index; WC, Waist Circumference; BMI, Body Mass Index; WWI, Waist-to-Weight index.

Compared to WWI and ABSI, BRI demonstrates slightly higher specificity, though it falls short of WC and BMI (Figure 3). However, considering the overall findings, BRI emerges as a highly promising indicator, showing a strong association with the occurrence of pOSA and consequently holding significant predictive value for OSA. Specific results from ROC analysis, including AUC, cutoff values, sensitivity, and specificity, are available in Supplementary Table 2.

## 4 Discussion

This study aimed to evaluate the association between several anthropometric indices-BRI, ABSI, WWI, WC, BMI and the prevalence of pOSA in a large, diverse population. Our findings indicate that BRI is significantly associated with pOSA, with this association remaining robust even after extensive adjustment for potential confounders. The identified threshold effect at a BRI of 4.3 suggests that individuals with higher BRI values are at substantially increased risk for pOSA, underscoring the importance of implementing targeted interventions for these high-risk populations. Interestingly, the ABSI inflection point of 8.2 reveals a positive association with pOSA prevalence on the left side of the inflection point; however, on the right side, a negative association emerges. This observation may explain the nonsignificant association between ABSI and pOSA occurrence in the fully adjusted model. Notably, BRI demonstrated superior predictive performance for pOSA risk compared to other indices such as ABSI and WWI, indicating that BRI could be a more effective tool for identifying individuals at risk for this condition.

Obesity is a recognized risk factor for OSA, with a notable correlation between the extent of obesity and the severity of the condition. Traditional anthropometric measures, such as BMI, WC, and WWI, are frequently utilized in clinical and research contexts to assess this relationship. Among these measures, BMI is the most commonly used due to its simplicity, but its effectiveness is often debated because it does not account for fat distribution (24). While WC partially mitigates this issue by addressing waist size, it fails to consider an individual’s height and weight, leading to inaccurate assessments of obesity prevalence, especially in individuals of varying heights. The WWI, calculated by dividing WC by body weight, has been found to have a positive correlation with fat mass, particularly in geriatric populations. Given these limitations, it is essential to identify the most accurate obesity indices for effective diagnosis and management of OSA.

Traditional obesity measures have been extensively studied in OSA. A prospective study involving 247 children found that BMI and WC were elevated in those with OSA, with strong correlations between these indices, inflammatory markers, and leptin (25). However, longitudinal studies indicate that while general obesity indices do not significantly correlate with OSA improvement, changes in visceral fat indices do (26). These findings may indirectly support our study’s conclusions. Nonetheless, the literature presents conflicting evidence. A meta-analysis encompassing 19 studies found no significant relationship between OSA and BMI, WC, or waist-hip ratio (27). Additionally, studies on adolescents and children revealed no connection between OSA and abdominal obesity, although central sleep apnea was related to increased abdominal fat levels (28). These inconsistencies may be attributed to variations in study populations, ethnicities, and OSA evaluation criteria.

Visceral obesity is a well- recognized risk factor for cardiovascular events and all-cause mortality. Given the challenges of directly measuring visceral fat, new AHIs like ABSI and BRI have been developed through algorithmic modeling. ABSI is more effective than both BMI and waist circumference in predicting all-cause mortality, but it is less reliable for forecasting chronic diseases (28, 29). BRI, which represents the body as an ellipse with height as the major axis and waist circumference as the minor axis, provides insights into fat distribution in obese individuals. The eccentricity of this ellipse defines the BRI, making it a more effective index for estimating abdominal obesity. The robust association between BRI and pOSA identified in this study highlights its potential as a valuable screening tool for pOSA.

Our findings align with previous research demonstrating the link between obesity-related metrics and pOSA. However, the present study adds to the literature by highlighting the superiority of BRI over other commonly used indices. While BMI has traditionally been the most widely used measure for assessing obesity-related health risks, our results suggest that BRI, which incorporates both body shape and fat distribution, may provide a more accurate reflection of pOSA risk. The comparative analysis highlighting that BRI has a higher predictive ability for pOSA than ABSI and WWI is particularly noteworthy. This suggests that BRI’s incorporation of both waist and height measurements offers a more detailed evaluation of body fat distribution, which is crucial in the development of pOSA. Research has demonstrated that BRI is superior to ABSI in predicting metabolic syndrome (30), hypertension and hyperuricemia (31). Moreover, the association between obesity and OSA is largely attributed to the accumulation of abdominal fat. This visceral fat exerts a greater influence on the upper airway than peripheral fat. These factors together elucidate why BRI is more effective than other indices in predicting pOSA.

The results of this study carry important clinical implications. Given the increasing prevalence of OSA and its associated health risks, there is a pressing need for effective screening tools. BRI, with its strong predictive ability and ease of calculation, could be integrated into routine clinical practice to pinpoint individuals at elevated risk for pOSA. Early identification and intervention are essential to mitigate the complications associated with pOSA, such as cardiovascular disease, metabolic disorders, and diminished quality of life.

The study’s strengths include its large and diverse sample size, as well as the use of a comprehensive set of covariates, which bolster the generalizability of our findings. Additionally, the application of advanced statistical methods allowed for a detailed exploration of the relationship between BRI and OSA, including the identification of non-linear associations. However, several limitations should be noted. First, the cross-sectional design limits the ability to draw causal inferences. Second, OSA diagnosis relied on self-reported data, which may be subject to reporting bias. Finally, while BRI appears to be a promising tool for predicting OSA, further research is required to validate these findings in different populations and to explore the potential of BRI in guiding clinical decision-making.

In conclusion, this study shows that BRI has a significant association with OSA prevalence and may provide advantages over other anthropometric measures in predicting OSA risk. Integrating BRI into clinical practice could improve early detection of OSA, especially in high-risk populations. Future research should aim to validate these results in longitudinal studies and investigate the potential of BRI as a foundation for targeted interventions to reduce the impact of OSA.

## Data Availability

Publicly available datasets were analyzed in this study. This data can be found at: https://www.cdc.gov/nchs/nhanes/.

## References

[1] Benjafield AV Ayas NT Eastwood PR Heinzer R Ip MSM Morrell MJ. Estimation of the global prevalence and burden of obstructive sleep apnoea: a literature-based analysis. Lancet Respir Med. (2019) 7:687–98. doi: 10.1016/S2213-2600(19)30198-5, PMID: 31300334 PMC7007763

[2] O'Connor GT Caffo B Newman AB Quan SF Rapoport DM Redline S. Prospective study of sleep-disordered breathing and hypertension the sleep heart health study. Am J Respir Crit Care Med. (2009) 179:1159–64. doi: 10.1164/rccm.200712-1809OC, PMID: 19264976 PMC2695498

[3] Abelleira R Zamarrón C Riveiro V Casal A Toubes ME Rábade C. Relationship between obstructive sleep apnea and type 2 diabetes mellitus. Med Clin (Barc). (2024) 162:363–9. doi: 10.1016/j.medcli.2023.11.014, PMID: 38220552

[4] Pérez-Carbonell L. Bashir S narrative review of sleep and stroke. J Thorac Dis. (2020) 12:S176–90. doi: 10.21037/jtd-cus-2020-002, PMID: 33214922 PMC7642629

[5] Li MF Sun ZR Sun HR Zhao GC Leng B Shen TQ. Paroxysmal slow wave events are associated with cognitive impairment in patients with obstructive sleep apnea. Alzheimers Res Ther. (2022) 14:14. doi: 10.1186/s13195-022-01153-x 36585689 PMC9801625

[6] Mehra R Benjamin EJ Shahar E Gottlieb DJ Nawabit R Kirchner HL. Association of nocturnal arrhythmias with sleep-disordered breathing - the sleep heart health study. Am J Respir Crit Care Med. (2006) 173:910–6. doi: 10.1164/rccm.200509-1442OC, PMID: 16424443 PMC2662909

[7] López-Padrós C Salord N Alves C Vilarrasa N Gasa M Planas R. Effectiveness of an intensive weight-loss program for severe OSA in patients undergoing CPAP treatment: a randomized controlled trial. J Clin Sleep Med. (2020) 16:503–14. doi: 10.5664/jcsm.8252, PMID: 32003737 PMC7161448

[8] Tuomilehto HPI Seppa JM Partinen MM Peltonen M Gylling H Tuomilehto JOI. Lifestyle intervention with weight reduction first-line treatment in mild obstructive sleep apnea. Am J Respir Crit Care Med. (2009) 179:320–7. doi: 10.1164/rccm.200805-669OC 19011153

[9] Wen X-L Wu B-Z Li Y Yi B Peng X. Analysis of the aerodynamic characteristics of the upper airway in obstructive sleep apnea patients. J. Dent. Sci. (2024) 19:329–37. doi: 10.1016/j.jds.2023.03.013, PMID: 38303889 PMC10829548

[10] Feng Y Keenan BT Wang S Leinwand S Wiemken A Pack AI. Dynamic upper airway imaging during wakefulness in obese subjects with and without sleep apnea. Am J Respir Crit Care Med. (2018) 198:1435–43. doi: 10.1164/rccm.201711-2171OC, PMID: 30040909 PMC6290952

[11] Heinzer RC Stanchina ML Malhotra A Fogel RB Patel SR Jordan AS. Lung volume and continuous positive airway pressure requirements in obstructive sleep apnea. Am J Respir Crit Care Med. (2005) 172:114–7. doi: 10.1164/rccm.200404-552OC 15817803 PMC2718445

[12] Drager LF Togeiro SM Polotsky VY Lorenzi G. Obstructive sleep apnea a Cardiometabolic risk in obesity and the metabolic syndrome. J Am Coll Cardiol. (2013) 62:569–76. doi: 10.1016/j.jacc.2013.05.045, PMID: 23770180 PMC4461232

[13] Molnar V Lakner Z Molnar A Tarnoki DL Tarnoki AD Kunos L. The predictive role of subcutaneous adipose tissue in the pathogenesis of obstructive sleep Apnoea. Life-Basel. (2022) 12:12. doi: 10.3390/life12101504 PMC9605212 36294937

[14] Vgontzas AN Papanicolaou DA Bixler EO Hopper K Lotsikas A Lin HM. Sleep apnea and daytime sleepiness and fatigue: relation to visceral obesity, insulin resistance, and hypercytokinemia. J Clin Endocrinol Metab. (2000) 85:1151–8. doi: 10.1210/jcem.85.3.6484, PMID: 10720054

[15] Zhang J Liang D Xu L Liu Y Jiang S Han X. Associations between novel anthropometric indices and the prevalence of gallstones among 6,848 adults: a cross-sectional study. Front Nutr. (2024) 11:11. doi: 10.3389/fnut.2024.1428488 PMC11298442 39104753

[16] Wu LD Kong CH Shi Y Zhang JX Chen SL. Associations between novel anthropometric measures and the prevalence of hypertension among 45,853 adults: a cross-sectional study. Front Cardiovasc. Med. (2022) 9:9. doi: 10.3389/fcvm.2022.1050654 PMC9669705 36407444

[17] Ding Z Zhuang Z Tang R Qu X Huang Z Sun M. Negative association between body roundness index and bone mineral density: insights from NHANES. Front Nutr. (2024) 11:11. doi: 10.3389/fnut.2024.1448938 PMC11340502 39176032

[18] Zhang XQ Ma N Lin QS Chen KN Zheng FJY Wu J. Body roundness index and all-cause mortality among US adults. JAMA Netw Open. (2024) 7:7. doi: 10.1001/jamanetworkopen.2024.15051 PMC11154161 38837158

[19] Krakauer NY Krakauer JC. A new body shape index predicts mortality Hazard independently of body mass index. PLoS One. (2012) 7:e39504. doi: 10.1371/journal.pone.0039504, PMID: 22815707 PMC3399847

[20] Zhang X Ye R Sun L Liu X Wang S Meng Q. Relationship between novel anthropometric indices and the incidence of hypertension in Chinese individuals: a prospective cohort study based on the CHNS from 1993 to 2015. BMC Public Health. (2023) 23:23. doi: 10.1186/s12889-023-15208-7 36879238 PMC9990350

[21] Cho H-W Chung W Moon S Ryu O-H Kim MK Kang JG. Effect of sarcopenia and body shape on cardiovascular disease according to obesity phenotypes. Diabetes Metab J. (2021) 45:209–18. doi: 10.4093/dmj.2019.0223, PMID: 32662256 PMC8024159

[22] Pan X Zhang X Wu X Zhao Y Li Y Chen Z. Association between non-high-density lipoprotein cholesterol to high-density lipoprotein cholesterol ratio and obstructive sleep apnea: a cross-sectional study from NHANES. Lipids Health Dis. (2024) 23:209. doi: 10.1186/s12944-024-02195-w 38965618 PMC11223298

[23] Strenth C Wani A Alla R Khan S Schneider FD Thakur B. Obstructive sleep apnea and its cardiac implications in the United States: an age-stratified analysis between young and older adults. J Am Heart Assoc. (2024) 13:e033810. doi: 10.1161/JAHA.123.033810, PMID: 38842290 PMC11255750

[24] Bray GA Beyond BMI. Beyond BMI. Nutrients. (2023) 15:2254. doi: 10.3390/nu15102254, PMID: 37242136 PMC10223432

[25] Bhatt SP Guleria R Kabra SK. Metabolic alterations and systemic inflammation in overweight/obese children with obstructive sleep apnea. PLoS One. (2021) 16:e0252353. doi: 10.1371/journal.pone.0252353, PMID: 34086720 PMC8177414

[26] Zhao XL Xu HJ Qian YJ Liu YP Zou JJ Yi HL. Abdominal obesity is more strongly correlated with obstructive sleep apnea than general obesity in China: results from two separated observational and longitudinal studies. Obes Surg. (2019) 29:2535–47. doi: 10.1007/s11695-019-03870-z, PMID: 31111342

[27] Cho JH Choi JH Suh JD Ryu S Cho SH. Comparison of anthropometric data between Asian and Caucasian patients with obstructive sleep apnea: a Meta-analysis. Clin Exp Otorhinolaryngol. (2016) 9:1–7. doi: 10.21053/ceo.2016.9.1.1, PMID: 26976019 PMC4792237

[28] Verhulst SL Schrauwen N Haentjens D Suys B Rooman RP Van Gaal L. Sleep-disordered breathing in overweight and obese children and adolescents: prevalence, characteristics and the role of fat distribution. Arch Dis Child. (2007) 92:205–8. doi: 10.1136/adc.2006.101089, PMID: 17041010 PMC2083395

[29] Ji M Zhang S. An R effectiveness of a body shape index (ABSI) in predicting chronic diseases and mortality: a systematic review and meta-analysis. Obes Rev. (2018) 19:737–59. doi: 10.1111/obr.12666, PMID: 29349876

[30] Anto EO Frimpong J Boadu WIO Tamakloe V Hughes C Acquah B. Prevalence of Cardiometabolic syndrome and its association with body shape index and a body roundness index among type 2 diabetes mellitus patients: a hospital-based cross-sectional study in a Ghanaian population. Front Clin Diabetes Healthc. (2021) 2:807201. doi: 10.3389/fcdhc.2021.807201 36994331 PMC10012128

[31] Li Y Zeng L. Comparison of seven anthropometric indexes to predict hypertension plus hyperuricemia among U.S. adults. Front Endocrinol. (2024) 15:1301543. doi: 10.3389/fendo.2024.1301543 PMC10958198 38524637

