# Supplementary material for: Association of Body Roundness Index and A Body Shape Index with Obstructive Sleep Apnea: insights from NHANES 2015–2018 data
Source: Front Nutr. 2024 Nov 18;11:1492673. doi: 10.3389/fnut.2024.1492673 (PMC11608980; doi:10.3389/fnut.2024.1492673)
Supplement: Supplementary file 1 [file Data_Sheet_1.docx]

Supplementary Material

# Supplementary Figures and Tables

Supplementary Figure 1. Smoothed curve fit between BMI and pOSA


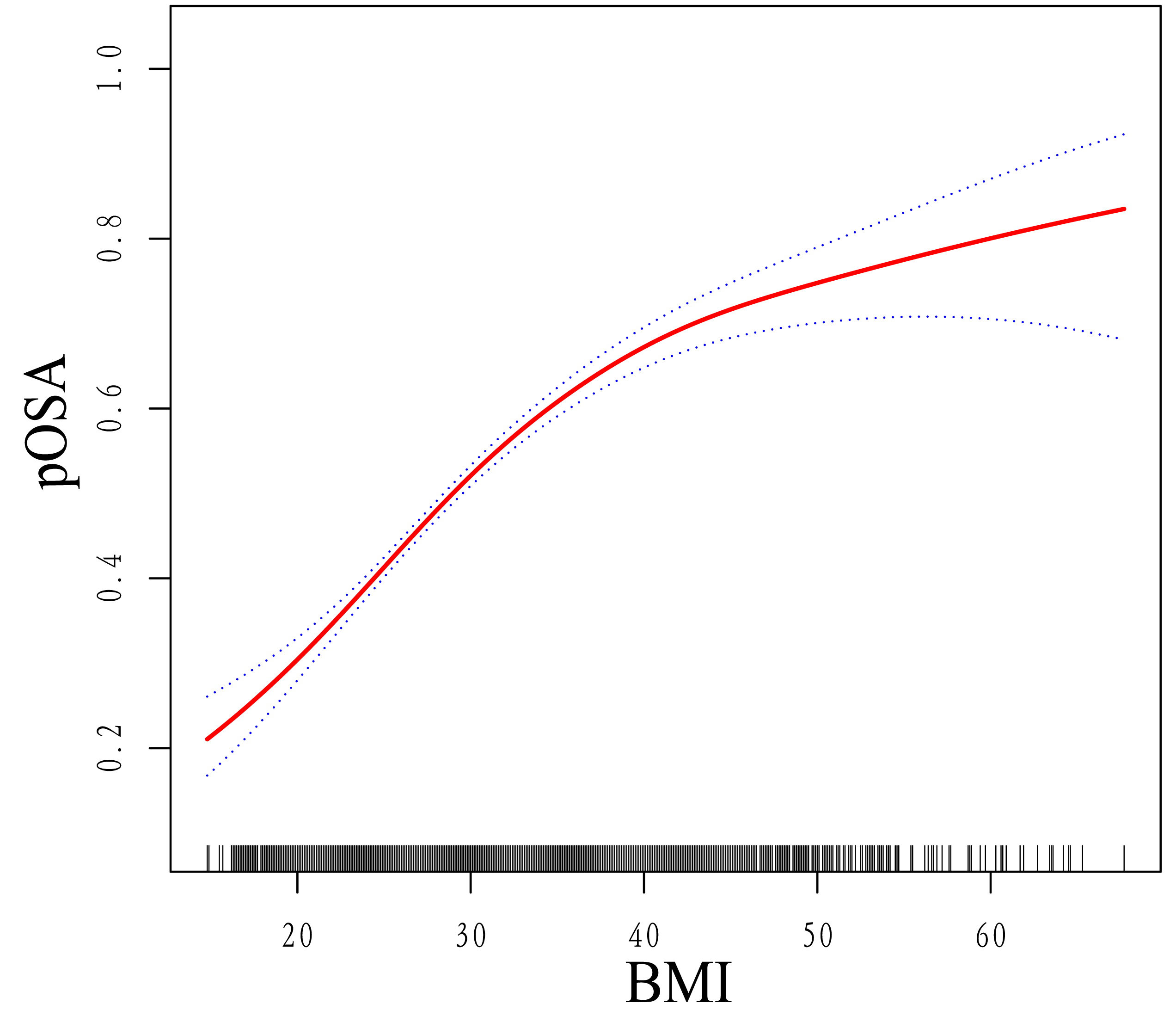


The blue bars show the fitted 95% confidence intervals (95% CI) and the fitted smoothed curves are shown in red. pOSA, Probable Obstructive sleep apnea; BMI, Body Mass Index

Supplementary Figure 2. Smoothed curve fit between WC and pOSA


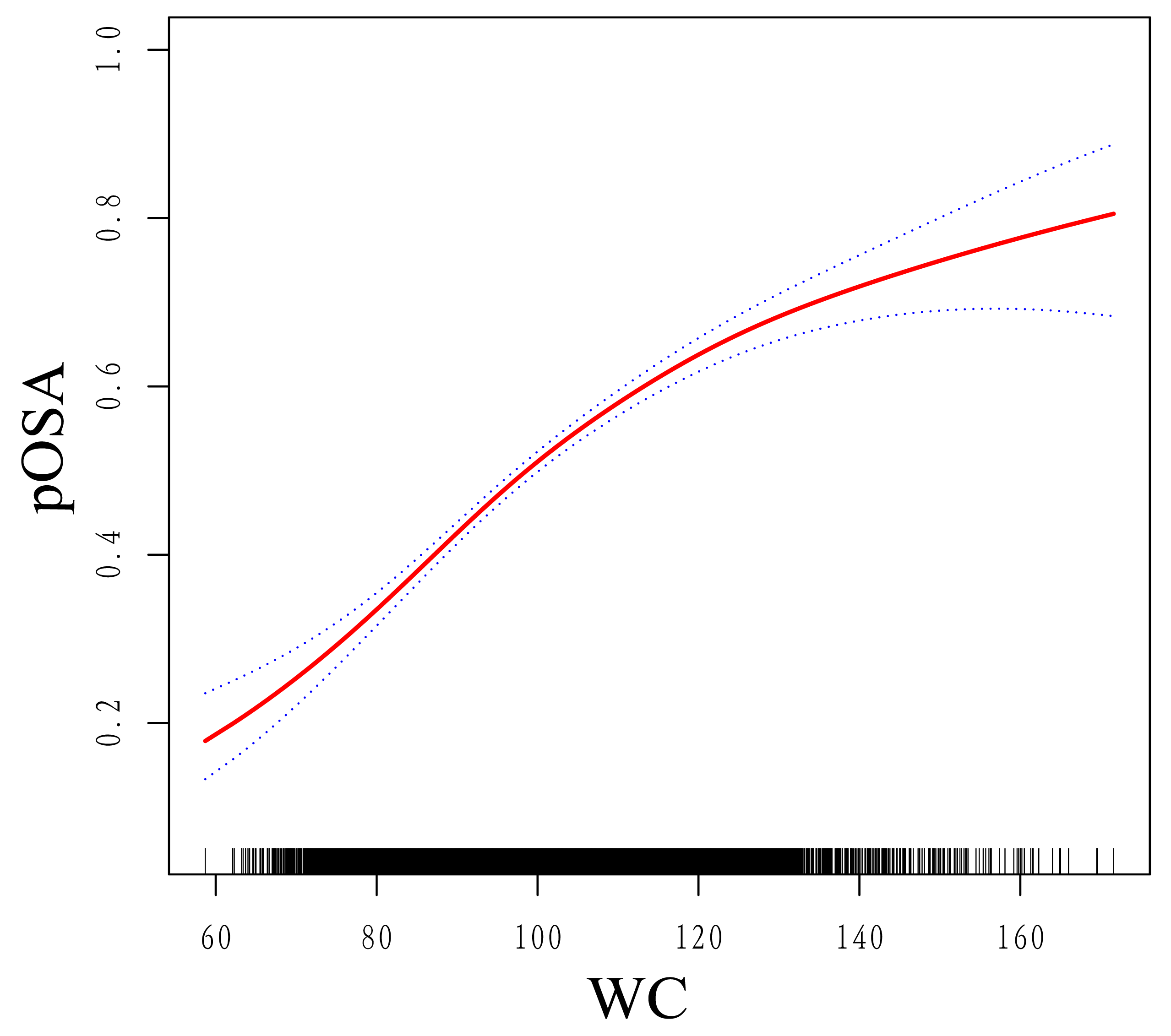


The blue bars show the fitted 95% confidence intervals (95% CIs) and the fitted smoothed curves are shown in red. pOSA, Probable Obstructive Sleep Apnea; WC, Waist Circumference

**Supplementary Figure 3. Smoothed curve fit between WWI and pOSA**


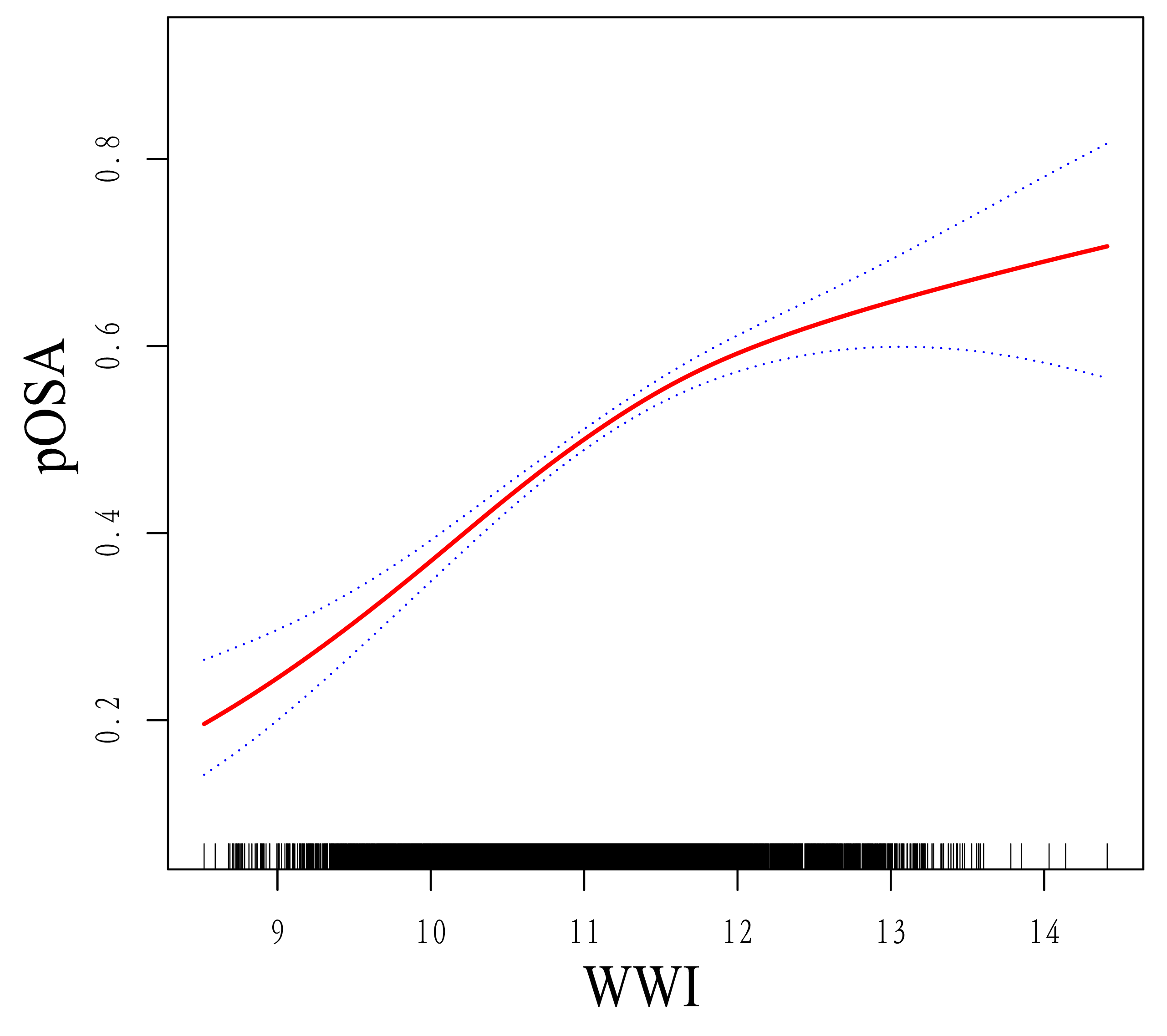


The blue bars show the fitted 95% confidence intervals (95% CIs) and the fitted smoothed curves are shown in red. pOSA, Probable Obstructive Sleep Apnea; WWI, Waist-to-Weight Index

**Supplementary Table 1. Covariates along with their descriptions or categorizations**


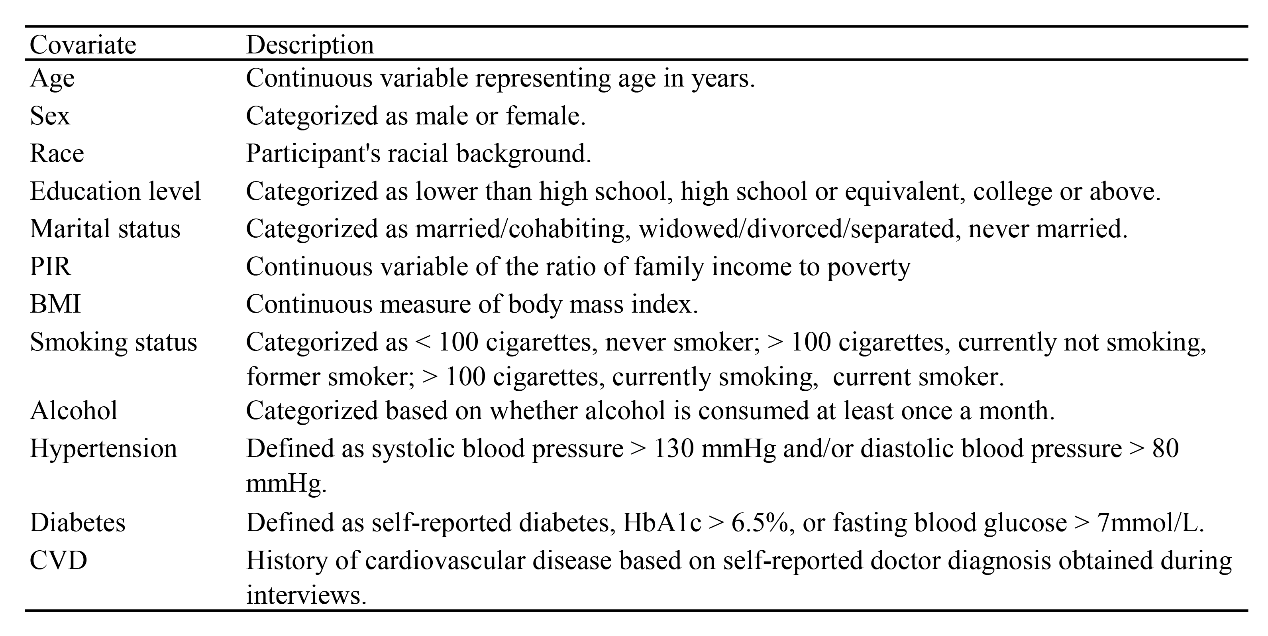


Covariates included in this study. PIR, Poverty Income Ratio; BMI, Body Mass Index; CVD, Cardiovascular Disease

**Supplementary Table 2. Area under the curve and cut-off values for AHIs as predictor of pOSA .**

**
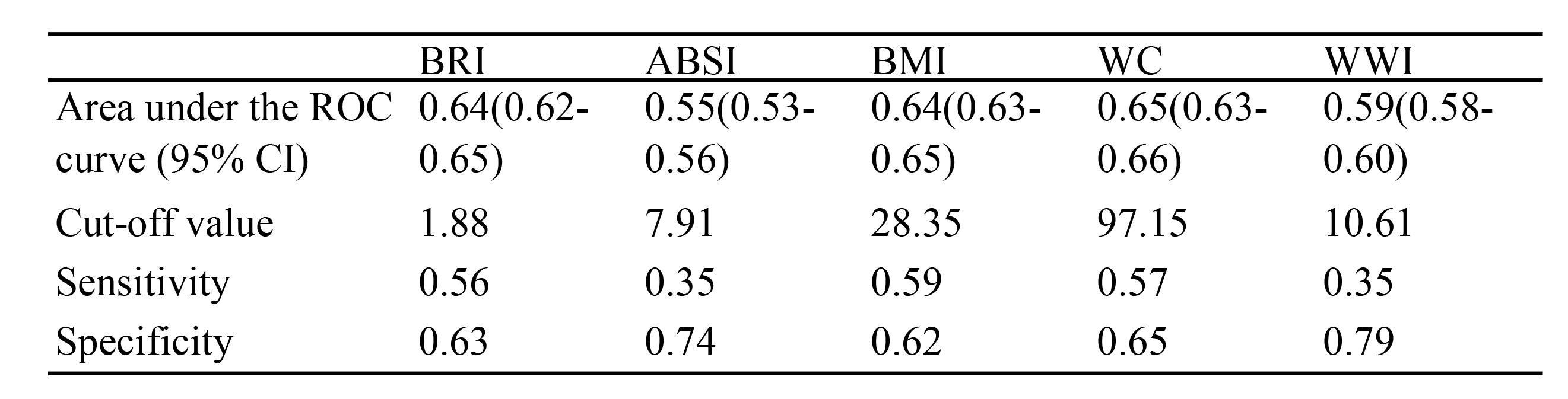
**

pOSA, Probable Obstructive sleep apnea; ROC, Receiver Operating Characteristics; CI, Confidence Interval; BRI, Body Roundness Index; ABSI, A Body Shape Index; BMI, Body Mass Index; WC, Waist Circumference;WWI, Waist-to-Weight Index
